# KnowPulse: A Web-Resource Focused on Diversity Data for Pulse Crop Improvement

**DOI:** 10.3389/fpls.2019.00965

**Published:** 2019-07-31

**Authors:** Lacey-Anne Sanderson, Carolyn T. Caron, Reynold Tan, Yichao Shen, Ruobin Liu, Kirstin E. Bett

**Affiliations:** Department of Plant Sciences, University of Saskatchewan, Saskatoon, SK, Canada

**Keywords:** legumes, pulses, web resource, diversity, genotypic data, phenotypic data

## Abstract

KnowPulse (https://knowpulse.usask.ca) is a breeder-focused web portal for pulse breeders and geneticists. With a focus on diversity data, KnowPulse provides information on genetic markers, sequence variants, phenotypic traits and germplasm for chickpea, common bean, field pea, faba bean, and lentil. Genotypic data is accessible through the genotype matrix tool, displayed as a marker-by-germplasm table of genotype calls specific to germplasm chosen by the researcher. It is also summarized on genetic marker and sequence variant pages. Phenotypic data is visualized in trait distribution plots: violin plots for quantitative data and histograms for qualitative data. These plots are accessible through trait, germplasm, and experiment pages, as well as through a single page search tool. KnowPulse is built using the open-source Tripal toolkit and utilizes open-source tools including, but not limited to, species-specific JBrowse instances, a BLAST interface, and whole-genome CViTjs visualizations. KnowPulse is constantly evolving with data and tools added as they become available. Full integration of genetic maps and quantitative trait loci is imminent, and development of tools exploring structural variation is being explored.

## Introduction

Legumes are immensely important in agricultural ecosystems with the legume family (Leguminosae) being second only to the grass family (Poaceae) in economic and nutritional value (Graham and Vance, 2003). Grain legumes, also known as “pulses,” are primarily marketed for human consumption and are a good source of dietary fiber, protein, slow-release carbohydrates, B vitamins, iron, copper, magnesium, manganese, zinc, and phosphorous (Tharanathan and Mahadevamma, 2003; Polak et al., 2015). They are also naturally low in fat, virtually free of saturated fat and cholesterol free (Polak et al., 2015). In recent years there has been an explosion of genome assemblies for legumes (Varshney et al., 2009, 2012, 2013; Schmutz et al., 2010, 2014; O'Rourke et al., 2014; Tang et al., 2014; Parween et al., 2015; Pandey et al., 2016). In addition, there has been a dramatic increase in sequence variation data (Kamfwa et al., 2015; Boutet et al., 2016; Moghaddam et al., 2016; Pandey et al., 2016; Gali et al., 2018; Ogutcen et al., 2018). In order to maximize the usefulness of this data, it should be curated with connections between phenotypic and genotypic data verified in a web resource which is friendly to both breeders and researchers.

Several legume-focused databases have been developed including Legume Information System (LIS; https://legumeinfo.org, Dash et al., 2015), Medicago truncatula Genome Database (http://www.medicagogenome.org, Krishnakumar et al., 2014), SoyBase (https://www.soybase.org/, Grant et al., 2009), PeanutBase (https://peanutbase.org; Dash et al., 2016), and Cool Season Food Legume Database (https://www.coolseasonfoodlegume.org/). While these resources are invaluable to their crop-specific and comparative communities, none provide the integration between germplasm, genotypic and phenotypic data to adequately develop the genetic markers useful in pulse breeding programs.

Over 100 plant and animal databases use Tripal (https://www.drupal.org/project/tripal; http://tripal.info/sites_using_tripal, Sanderson et al., 2013), an open-source, highly customizable toolkit providing efficient development of biological web portals. Tripal extends the popular Drupal content management system (CMS). Use of a CMS enables developers to focus on the specific needs of their community without the overhead of user and security management, or the database schema design frequently associated with web portal development. Tripal's use of the Generic Model Organism Database (GMOD) Chado schema (Mungall and Emmert, 2007) provides flexible support for biological data, while facilitating the exchange of data and expertise among Tripal sites through common infrastructure.

KnowPulse, a breeder-focused web portal, was first released in 2010 to serve the pulse breeders at the University of Saskatchewan. There is a focus on common bean, chickpea, field pea, lentil and faba bean, as these are the crops of interest in their program. KnowPulse is built using Tripal, with the purpose of serving as a reliable data storage solution with metadata preservation. It has since evolved into a public resource by housing a large number of continually expanding datasets focused on genetic variation. We describe the novel genetic variation display and tools of KnowPulse below to inform the greater legume community.

## Materials and Methods

### Datasets

KnowPulse houses data for chickpea, dry bean, field pea, lentil, and faba bean. The magnitude of all data is summarized by type (e.g., germplasm, genotypes, phenotypes) on the home page. There is information on Genebank accessions and University of Saskatchewan cultivars. Users can access a number of genotypic (i.e., genetic markers, sequence variants, and genotypic calls) and phenotypic (i.e., traits, experiments, and measurements) datasets. Lastly, the pre-release genomic sequence information for *Lens culinaris* is available through the web portal by request. In an effort to provide researchers with data as soon as possible, KnowPulse houses unpublished data. However, all data is required to have a long-term data management plan ensuring integrity and availability.

### Implementation

KnowPulse uses Drupal 7 (https://www.drupal.org/), an open-source enterprise-level content management system, and Tripal 3, which extends Drupal for biological data. The modular PHP framework provided by Drupal and Tripal allows KnowPulse to use community-contributed extensions and an advanced administrative interface to speed up development time and provide more functionality to users. The core Tripal modules power the ontology-driven content pages (e.g., genetic markers, germplasm accessions, research projects), content-type specific searches and semantic web-ready web services for all content. Customized displays were developed through extension modules. The entire technology stack is open-source and all extension modules are publicly available on GitHub and open to collaboration (https://uofs-pulse-binfo.github.io/our-modules/).

All data, excluding the BLAST databases, are stored in a single PostgreSQL instance using the Drupal schema and GMOD Chado schema (Mungall and Emmert, 2007) for web-related data and biological data, respectively. PostgreSQL constraints and data type checking ensure data integrity and standards compliance. For example, genotypic data must be linked to the germplasm assayed, the experiment, and the genetic marker including assay information. Well-chosen indices and materialized views mitigate any performance issues incurred by use of a relational database by speeding up queries. This combination allows us to meet the speed and data integrity needs of the user.

### Permissions and Accessibility

KnowPulse acts as both a public data portal and a private breeding program management system. All the functionality described herein is publicly available unless otherwise stated. Since KnowPulse provides access to pre-publication data, you may find restrictions on download for specific datasets and watermarked charts. Private data and tools can be accessed via a user account with specific permissions. If you need access to private data for your research, please contact Dr. Kirstin Bett, corresponding author, with an explanation and in most cases we will be happy to collaborate with you.

## Results and Discussion

### Genomic Variation

**Figure 1:**
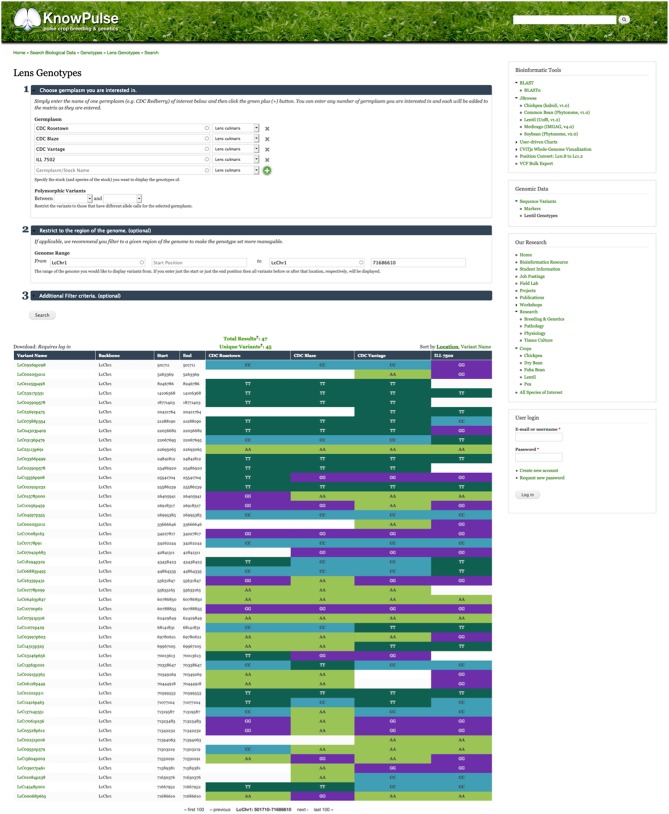
Germplasm by variant genotype matrix. This screenshot shows the genotype matrix for CDC Rosetown, CDC Blaze, CDC Vantage, and ILL 7502 restricted to the beginning of LcChr1. The form near the top provides additional filter options while the color-coded table below shows the allele calls for each known variant. Researchers can use this tool to inspect the genotypes of a region of interest (e.g., QTL region) for their germplasm set. This tool can be accessed in the right side menu under Genomic Data > Sequence Variants > Lentil Genotypes.

In the genomic context, genotypic data are particularly important in KnowPulse. These data are used by researchers for marker development and association studies with the ultimate goal of facilitating pulse crop breeding. KnowPulse provides a germplasm-by-variant genotype matrix for researchers to explore genotypic data for their germplasm set (Figure 1). Since genotypic datasets are increasingly expanding, this tool provides filter options including experiment, variant list, genomic position, marker or variant type, and pairwise polymorphisms. Additionally, if the data is overwhelming to analyze within the browser, users can request permission to downloaded it via KnowPulse in a variety of formats (e.g., comma-separated values, hapmap).

**Figure 2:**
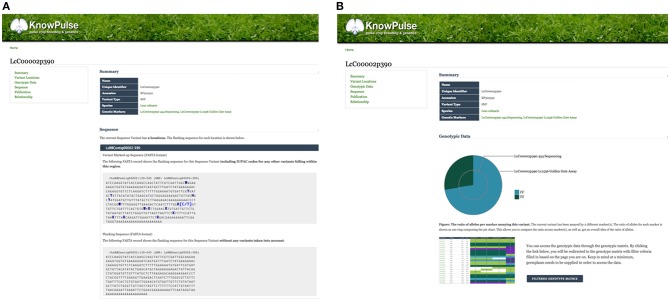
Genetic Marker and Sequence Variant pages on KnowPulse. Genetic Marker pages **(A)** describe the actual marker assay. This screenshot shows the flanking sequence of the marker with variants indicated by their IUPAC codes. Sequence Variant pages **(B)** describe a position in the genome and can be used for comparison of multiple markers. In the screenshot, the pie chart shows the ratio of observed alleles compared between a 454 Sequencing and Lc1536 Golden Gate marker. These pages can be accessed via the sequence variant or genetic marker search respectively under Genomic Data in the right side menu.

Sequence variants and genetic markers are each represented with their own pages in KnowPulse. Sequence variant pages list all the markers available for a given genomic position, whereas genetic marker pages provide details for a specific marker assay. This distinction allows researchers to evaluate genotypes in context of the assay. Additionally, genetic marker pages pinpoint the location of the variant on each available genome assembly. More advanced features include: the flanking sequence with additional known variants indicated using their IUPAC codes, a pie chart summarizing the allele calls recorded, and a link to the genotype matrix to access specific calls for germplasm of interest (Figure 2A). Sequence variant pages reveal similar information with the context of all markers for that variant for comparison (Figure 2B).

**Figure 3:**
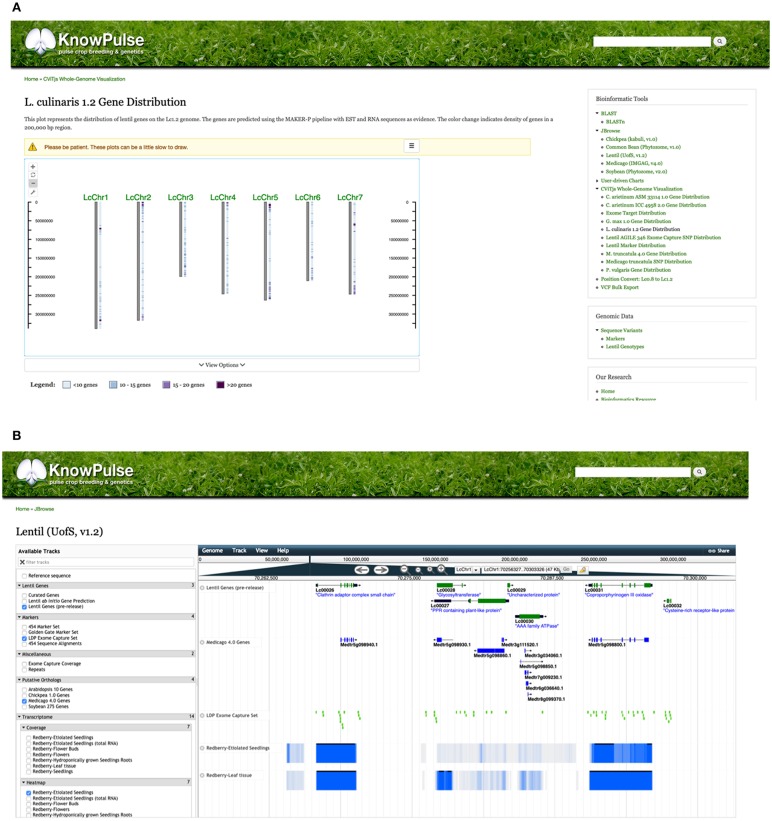
KnowPulse Genome Browsing Tools: CViTjs and JBrowse. CViTjs **(A)** is a whole-genome graphical viewer showing each chromosome with a track beside it summarizing data (https://github.com/LegumeFederation/cvitjs). In this case, the CViTjs plot is summarizing the distribution of genes as a heatmap track. JBrowse **(B)** shows a specific region of the genome with the ability to scroll left or right (Buels et al., 2016). Many tracks are supported, including gene models, putative homologous genes, genetic markers and RNAseq results as shown here on the Lentil v1.2 JBrowse. These tools can be accessed through the right side menu under Bioinformatics Tools.

A number of tools which provide further context to these genetic markers through whole-genome visualizations include CViTjs (https://github.com/LegumeFederation/cvitjs) and JBrowse (Buels et al., 2016). CViTjs provides whole-genome views of specific datasets such as gene and genetic marker distribution. These are available on KnowPulse for chickpea, common bean, lentil, soybean, and medicago (Figure 3A). CViTjs charts allow researchers to see broad trends across the genome; whereas, JBrowse instances are highly suitable for graphical browsing of a specific region of interest. KnowPulse has JBrowse instances for kabuli chickpea (v1.0, Varshney et al., 2013), common bean (v1.0, Schmutz et al., 2014), lentil (v1.2, Ramsay et al., 2014), soybean (v2.0, Schmutz et al., 2010), and medicago (v4.0, Tang et al., 2014) with tracks for gene sets, genetic markers, and putative orthologs from related species (Figure 3B).

Tripal BLAST (https://www.drupal.org/project/tripal_blast) provides sequence alignment searches for users with a region of interest but no prior information about its location in hosted genome assemblies. In KnowPulse, users can BLAST against pulse-specific datasets such as genome and transcript assemblies for crops (i.e., chickpea, common bean, field pea, and lentil), related wild species, and model legume species (i.e., soybean, lotus, medicago). The user simply enters their sequence in the search box, selects the dataset to BLAST against and clicks BLAST, which uses NCBI BLAST+ command-line tools (Camacho et al., 2009) to perform the search. The results are then displayed in a table with links to the appropriate JBrowse.

### Phenomic Variation

With our focus on variation data, phenomics is a very important component of KnowPulse. Not only are phenotypic data used for association studies and marker discovery, they are also used for breeding activities such as germplasm selection and identification. As such, visualizations focus on the distribution of phenomic data, often in reference to specific germplasm and between site-years within an experiment.

**Figure 4:**
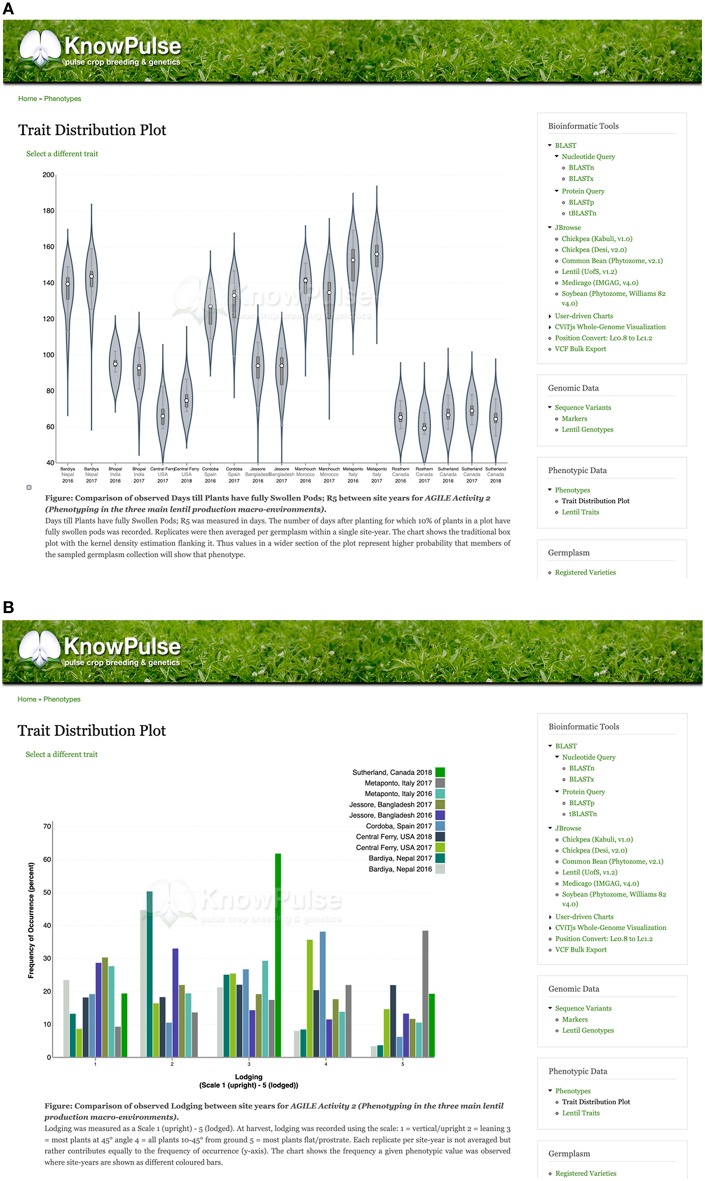
Trait distribution plots summarizing phenotype data. Quantitative phenotypic data **(A)** is shown as a violin plot with site-year labeled by the x-axis and observed values labeled by the y-axis. This allows researchers to see the data structure (i.e., median, interquartile range, and 95% confidence interval) and distribution per site year. Qualitative phenotypic data **(B)** is shown as a multi-series histogram with each series representing a site-year and the observed phenotypes defined on the x-axis. The quantity of germplasm exhibiting each phenotype is shown on the y-axis allowing researchers to evaluate how prevalent a phenotype is in their population. These plots can be accessed via the trait distribution plot tool under Phenotypic data in the right side menu, as well as through trait, germplasm, and project pages with associated phenotypic data.

KnowPulse provides trait distribution plots to summarize phenotypic data for a given experiment. Data from different site-years are stored separately but averaged across replicates. For quantitative data, violin plots are used to demonstrate data structure (i.e., median, interquartile range, and 95% confidence interval) and distribution. The x-axis labels each site-year, whereas the y-axis labels the observed values for the given trait (Figure 4A). Qualitative data is summarized with histograms which consist of a series for every site-year (Figure 4B). In both plot types, the phenotypic value for a given germplasm can be highlighted within the context of the larger dataset. This proves quite helpful in breeding programs to provide additional data for selections, highlight potential planting errors, and plan crosses.

Trait distribution plots can be accessed in a number of different ways. Plots are found on all associated trait, germplasm, and experiment pages. There is also a tool which allows users to generate their own plots based on KnowPulse-housed data. This kind of integration ensures that the system is intuitive to all users. Context and summaries for the trait, experiment or germplasm being viewed is also provided.

**Figure 5:**
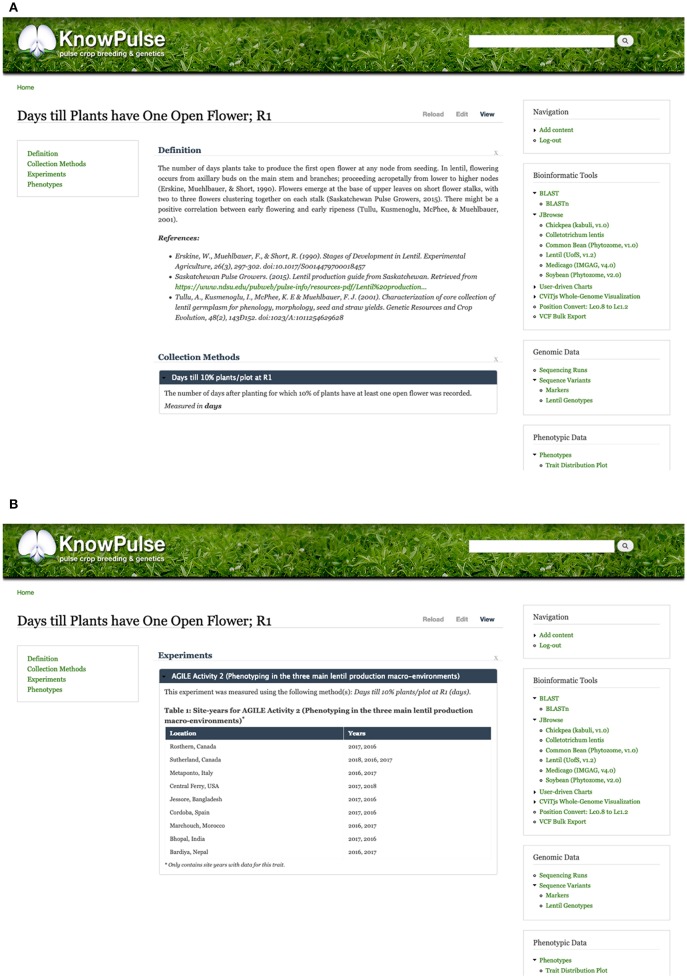
KnowPulse trait pages. Each trait page describes the methods and units used for collecting the data **(A)** and lists all experiments the trait was measured in **(B)**. This information provides context to help researchers better interpret the data. The trait distribution plot shown in Figure 4 is also available on each trait page. Trait pages can be accessed via the crop-specific trait search in the right side menu under phenotypic data.

Additionally, trait pages in KnowPulse contain an overview describing the trait, linking it to ontologies and describing the methodology used for data collection (Figure 5A). Experiments in which the traits were measured are listed, along with information on the number of associated site-years (Figure 5B). Traits can be searched for by keyword and filtered by a minimum number of site-years or germplasm.

### Germplasm

**Figure 6:**
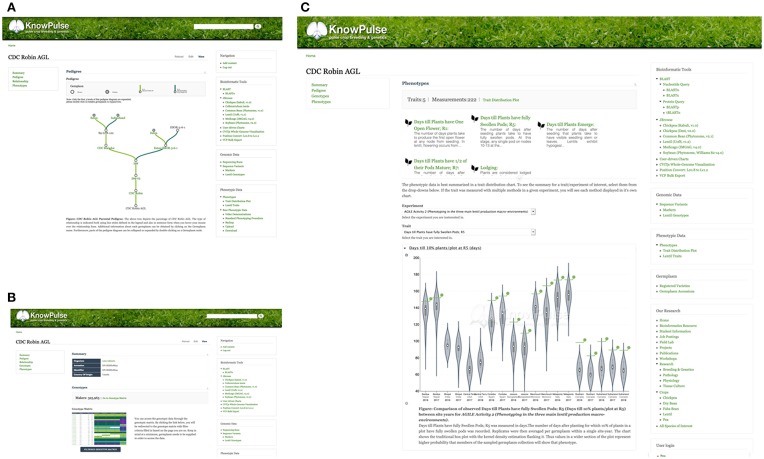
KnowPulse Germplasm pages. Each germplasm page describes the material and data collected for it. The parental pedigree **(A)**, is shown graphically with each parent having a link for further information. The magnitude of genotypic **(B)** and phenotypic **(C)** data is indicated on the page. Specifics for a particular experiment can be accessed through a link to the genotype matrix and trait distribution plot for genotypes and phenotypes, respectively. Germplasm pages can be accessed via the registered varieties and germplasm accession searches in the right side menu under germplasm.

At the core of KnowPulse are the germplasm collections including both public diversity panels and private crossing blocks. Germplasm pages contain all metadata stored in KnowPulse (e.g., origin, name, synonyms, accessions, known parents). Known pedigrees are displayed in a tree diagram with collapsible nodes (Figure 6A). The magnitude of genotypic data available for that individual is indicated, followed by a quick marker search and a link to the genotype matrix (Figure 6B). Similarly, the phenotypic data section contains an indication of magnitude, trait quick search, and access to the trait distribution plot (Figure 6C). Specialized searches depending on the type of germplasm (e.g., accessions vs. breeding material) with specific filter criteria are available. For example, accessions can be searched by name or accession; whereas, breeding material can also be restricted by crossing block.

## Conclusion

As a breeder-focused resource, KnowPulse emphasizes germplasm information and variation. Both genotypic and phenotypic data are supported with rich visualizations and detailed pages. Future enhancements include support for genetic maps and quantitative trait loci (QTL), as well as enhanced displays for exploring structural variation. KnowPulse is continually updated as new data become available.

## Data Availability

All datasets analyzed for this study are included in the manuscript and/or the supplementary files.

## Author Contributions

L-AS provided the initial concept and design for the resource with extensive input from KB. L-AS and CC wrote the manuscript. L-AS, CC, RT, and YS contributed significantly to the development of the resource. CC, RL, and L-AS curated the data and populated the resource. KB contributed a lot of the data held in the resource. All authors contributed to manuscript revision, read and approved the submitted version.

### Conflict of Interest Statement

The authors declare that the research was conducted in the absence of any commercial or financial relationships that could be construed as a potential conflict of interest.
